# Multidrug-resistant Staphylococcus hominis endophthalmitis after cataract surgery: A case report

**DOI:** 10.1016/j.idcr.2026.e02508

**Published:** 2026-02-01

**Authors:** Athina Droulia, Foteini Gyftaki, Panagiotis Stavrakas, Vasileios Papastavrou, Evripidis Sykakis, George Margetis, Athanasios Margetis, Anastasia Tsiogka

**Affiliations:** a Department of Ophthalmology, National and Kapodistrian University of Athens, School of Medicine, Evgenideion Hospital, Athens, Greece; b Department of Ophthalmology, General Hospital of Athens "G. Gennimatas", Athens, Greece; c Department of Anterior Segment & Glaucoma, Eye Day Clinic, Athens, Greece; d Emmetropia Mediterranean Eye Institute, Heraklion, Greece; e Department of Ophthalmology, Tzaneio General Hospital of Piraeus, Athens, Greece; f Department of Ophthalmology, Darlington Memorial Hospital, UK

**Keywords:** Endophthalmitis, Cataract surgery, Staphylococcus hominis, Multidrug resistance

## Abstract

**Introduction:**

Postoperative endophthalmitis is a rare but vision-threatening complication of cataract surgery. Multidrug-resistant organisms present additional therapeutic challenges.

**Case presentation:**

We report a 52-year-old man who underwent uncomplicated phacoemulsification for posterior subcapsular cataract. On the first postoperative day, visual acuity was 8/10, but inflammation gradually appeared without the classic signs of endophthalmitis. On the fifth postoperative day, the patient's vision deteriorated significantly, reaching 1/10, accompanied by a strong inflammatory reaction in the anterior chamber. A pars plana vitrectomy was performed and vancomycin and ceftazidime were administered intravitreally. *Staphylococcus hominis*, resistant to most common antibiotics but sensitive to gentamicin, was isolated from the vitreous culture. The medication was modified accordingly, with topical gentamicin and oral doxycycline, under daily close monitoring. One month later, cystoid macular edema appeared, which was successfully treated with acetazolamide, topical corticosteroids, and bromfenac. Three months after surgery, the patient's vision had returned to 9/10 without any permanent damage.

**Conclusion:**

Early surgical intervention, culture-guided therapy, and intensive follow-up can preserve vision even in postoperative endophthalmitis due to multidrug-resistant organisms.

## Introduction

Postoperative endophthalmitis is a rare but severe complication of cataract surgery, with contemporary studies reporting an incidence below 0.1 % [1]. Gram-positive organisms, particularly coagulase-negative staphylococci such as *Staphylococcus epidermidis* are the most isolated pathogens [2]. Despite established empirical therapy with intravitreal vancomycin and ceftazidime, based on The Endophthalmitis Vitrectomy Study, emerging multidrug resistance poses a therapeutic challenge and may compromise visual outcomes [2], [3], [4].

Among coagulase-negative staphylococci, Staphylococcus hominis is a rare cause of endophthalmitis, with only a few cases reported in the literature [5], [6]. These cases highlight the potential for atypical clinical presentations, resistance to standard antibiotics, and variable visual prognosis.

We report a case of multidrug-resistant *S. hominis* endopthalmitis following uneventful phacoemulsification in 52- year-old man, underscoring the importance of early vitrectomy, culture guided antimicrobial therapy and close follow-up in preserving vision.

## Case presentation

A 52-year-old man presented to the office with blurred vision. The patient's best-corrected visual acuity was 8/10 in the right eye with a correction of + 1.50 sph and 6/10 in the left eye with the same correction. Slit-lamp examination revealed posterior subcapsular cataracts in both eyes (more advanced picture in the left eye). Personal and family medical history were unremarkable. The patient was a smoker and reported occasional alcohol consumption. He was not taking any chronic medication and had no known allergies.

He underwent uncomplicated phakic resection with intraocular lens placement in the left eye. During the postoperative course, on the first day the vision was 8/10, with mild discomfort and a small amount of residual material behind the lens. On the third day, redness of the eye was observed, without change in visual acuity. On the fourth day, blurred vision appeared, with a decrease in visual acuity to 4/10 and a reaction in the anterior chamber (Tyndall +++). On the fifth day, vision decreased further to 1/10, with a pronounced inflammatory reaction (Tyndall ++++), circular injection, but without the presence of pus, eyelid edema or secretions.

The initial postoperative inflammation was considered atypical because of the absence of classic signs of endophthalmitis such as hypopyon, lid edema, severe pain, or purulent discharge. However, the rapid deterioration of visual acuity from 8/10 on day 3–1/10 on day 5, together with a marked increase in anterior chamber reaction (Tyndall ++++), strongly suggested infectious endophthalmitis.

**Fig. 1:**
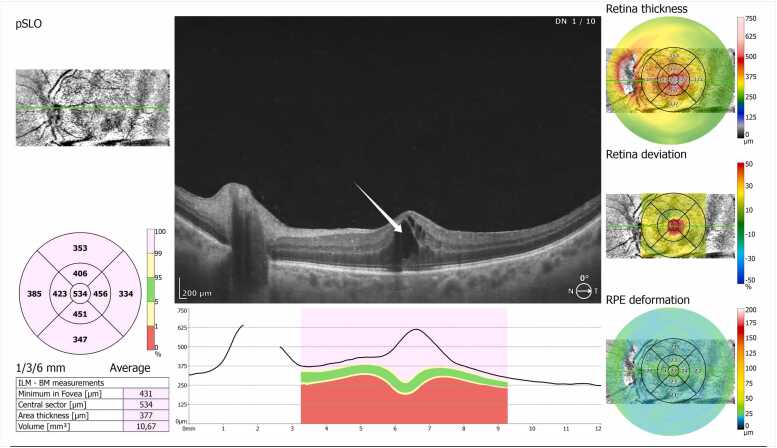
Macular OCT demonstrates cystoid macular oedema, evidenced by intraretinal hyporeflective cystic spaces predominantly involving the inner nuclear and outer plexiform layers, with associated macular thickening (arrow) one month after treatment for endophthalmitis.

The patient underwent pars plana vitrectomy, vitreous samples were received for microbiological cultures and empirical intravitreal vancomycin (1 mg/0.1 mL) and ceftazidime (2.25 mg/0.1 mL) were administered. The vitrectomy technique included typical 23 G pars plana approach after anterior chamber washout. Intravitreal samples were taken via a vitrector and manual aspiration from a syringe before infusion. Following that, standard vitrectomy ensued with antibiotics. Microbiological analysis of the vitreous sample yielded *Staphylococcus hominis*, demonstrating multidrug resistance with susceptibility only to gentamicin (Table 1). Based on the culture results, the antimicrobial regimen was revised: topical gentamicin 0.5 %/ dexamethasone 0.1 % eye drops were administered six times daily for the first three weeks in combination with oral doxycycline 100 mg twice daily for ten days. Once the clinical condition stabilized, the dosage was reduced to four times daily and maintained at this frequency without further tapering because month after surgery, the patient developed cystoid macular edema **(**Fig. 1**)**. Oral acetazolamide (250 mg three times daily) was initiated combined with topical gentamicin 0.5 %/ dexamethasone 0.1 % eye drops (one drop six times daily) and bromfenac 0.9 mg/mL (one drop three times daily). One month after the appearance of CMO, follow-up examinations showed complete resolution of the edema and absence of intraocular inflammation; therefore, acetazolamide was discontinued, and topical gentamicin 0.5 %/dexamethasone 0.1 % was gradually tapered over one month. At three months after surgery, the patient’s visual acuity had recovered to 9/10 without correction, and no structural or functional sequelae were detected on ocular examination (Table 2).

**Table1:** Antimicrobial susceptibility profile of the *Staphylococcus hominis* ssp. hominis isolate recovered postoperatively from the 52-years-old patient’s vitreous sample following pars plana vitrectomy.

Antimicrobial	MIC (mg/L)	Interpretation
Cefoxitin Screen	POS	**+**
Benzylpenicillin	-	-
Oxacillin	> 2	R
Gentamicin	1	S
Tobramycin	> 8	R
Ciprofloxacin	-	R
Levofloxacin	> 4	R
Moxifloxacin	4	R
Inducible Clindamycin Resistance	NEG	**-**
Erythromycin	> 4	R
Clindamycin	> 4	R
Linezolid	> 4	R
Teicoplanin	≤ 0.5	S
Vancomycin	> 16	R
Tetracycline	≤ 1	S
Tigecycline	≤ 0.12	S
Fosfomycin	> 64	R
Nitrofurantoin	-	-
Fusidic Acid	> 16	R
Rifampicin	1	I
Trimethoprim/Sulfamethoxazole	80	I

MIC = minimum inhibitory concentration, defined as the lowest concentration of an antimicrobial agent that inhibits visible growth of the microorganism in vitro. The interpretation categories follow EUCAST criteria: *S (susceptible)* indicates that the isolate is likely to be inhibited by the usually achievable concentrations of the agent when used with standard dosing; *I (intermediate)* denotes reduced susceptibility, where clinical efficacy may be uncertain and dependent on dosage or infection site; *R (resistant)* means that the isolate is not expected to be inhibited by the drug at standard concentrations, and clinical failure is likely.

**Table 2:** Timeline of clinical findings, microbiology, management, and visual outcomes of the 52-years-old patient with postoperative endophthalmitis.

Timepoint	Event	Clinical findings/Management
Pre-op	Diagnosis of bilateral posterior subcapsular cataracts (worse in LE)	VA: RE 8/10, LE 6/10 (+1.50 sph)
Day 0	Phacoemulsification, left eye	Intraocular lens implanted
Day 1	First postoperative visit	VA 8/10, mild discomfort, residual posterior capsular element noted
Day 3	Postoperative day 3	Redness, VA stable at 8/10
Day 4	Postoperative day 4	VA decreased to 4/10, blurred vision, anterior chamber reaction (Tyndall +++)
Day 5	Postoperative day 5	VA 1/10, Tyndall ++++, ciliary injection, no hypopyon; pars plana vitrectomy performed, intravitreal vancomycin & ceftazidime administered
Post-op cultures	Vitreous sample	Growth of MDR *Staphylococcus hominis*, resistant to multiple agents, sensitive only to gentamicin
Following days	Adjusted therapy	Topical gentamicin, oral doxycycline, close daily follow-up
1 month	Complication	Cystoid macular edema → treated with acetazolamide, topical dexamethasone, bromfenac
3 months	Outcome	VA 9/10 uncorrected, no residual pathology

VA = visual acuity; LE = left eye;, RE=right eye; sph = spherical correction; MDR = multidrug-resistant.

## Discussion

Endophthalmitis after cataract surgery is a rare but vision-threatening complication, most commonly caused by coagulase-negative staphylococci such as Staphylococcus epidermidis [1], [2]. Among these organisms, *Staphylococcus hominis* is an uncommon pathogen, with only sporadic cases reported to date. Available reports have included both acute and chronic postoperative infections as well as trauma-related cases, and visual outcomes have been variable [5], [6], [7], [8].

Iyer et al. reported a case of delayed endophthalmitis following cataract surgery, in which *S. hominis* was isolated. The infection had a chronic course and visual recovery was limited [4]. Won and Kim reported acute postoperative endophthalmitis due to vancomycin-resistant *S. hominis*; despite intensive treatment with vitrectomy and intraocular lens removal, the patient's final visual acuity remained low (20/400) three months later [5]. More recently, Aykut et al. reported two new cases, one after intravitreal injection and the other after an undiagnosed trauma, confirming the organism's potential to cause serious ocular infections [6]. Chronic forms have also been reported after penetrating wounds with retained foreign bodies [7].

In contrast to what has been described in previous reports, our patient presented with an earlier and more unusual form of endophthalmitis, with intense inflammation after surgery but without its typical signs, such as hypopyon, eyelid edema, or discharge. The rapid deterioration of vision and the increasing inflammation led to the decision for immediate vitrectomy, which proved to be crucial for saving vision.

Vitreous culture showed *Staphylococcus hominis*, resistant to most common antibiotics, including vancomycin, but sensitive to gentamicin. The broad antimicrobial resistance profile observed in this case can be explained by known genetic mechanisms in coagulase-negative staphylococci. In particular the presence of the mecA gene, which encodes the altered penicillin- binding protein PBP2a, confers resistance to beta-lactam antibiotics and had been increasingly identified in *S.hominis* isolates. Additional resistance determinates, including genes affecting macrolides, tetracyclines and aminoglycosides, has been reported, contributing to the limited efficacy of standard empirical regimens [9]. After adjusting the treatment — topical gentamicin and oral doxycycline — the condition improved markedly. Close, daily monitoring helped in the early recognition and successful treatment of cystic macular edema, preventing further deterioration.

The present case indicates that a postoperative inflammation without typical features does not exclude the presence of infection. On-time surgical and culture-guided antibiotic treatment are important, especially when resistant microorganisms are involved. Continuous monitoring ensures early recognition and effective management of complications, ultimately emphasizing the importance of vigilance and tailored treatment strategies in rare, multidrug-resistant postoperative endopthalmitis.

## Conclusion

Postoperative endophthalmitis can occur even without the classic clinical signs and be caused by rare, resistant bacteria such as *Staphylococcus hominis*. Early recognition, cultured- guided antimicrobial therapy and intensive monitoring are crucial for preserving vision in rare, multidrug - resistant endopthalmitis.

## Ethical statement

This case report was conducted in accordance with the ethical standards of the responsible institutional and national research committees and with the principles of the Declaration of Helsinki. Written informed consent was obtained from the patient for participation in the study and for the publication of this case report and any accompanying images. Patient anonymity has been preserved, and no identifying information is included in the manuscript.

## CRediT authorship contribution statement

**Anastasia Tsiogka:** Writing – review & editing, Visualization, Validation, Supervision, Methodology, Investigation, Formal analysis, Data curation, Conceptualization. **DROULIA ATHINA:** Writing – original draft, Visualization, Validation, Project administration, Methodology, Investigation, Data curation. **Foteini Gyftaki:** Validation, Methodology. **Panagiotis Stavrakas:** Visualization, Project administration, Methodology, Investigation. **George Margetis:** Project administration, Methodology. **Athanasios Margetis:** Project administration, Methodology. **Vasileios Papastavrou:** Writing – review & editing, Project administration, Methodology, Investigation, Data curation. **Evripidis Sykakis:** Visualization, Validation, Methodology.

## Patient consent

Written informed consent was obtained from the patient for publication of this case report and any accompanying images.

## Funding

The publication of this article was supported by the 10.13039/501100005187National and Kapodistrian University of Athens. The funder had no role in the study design, data collection, analysis, interpretation of data, or in the writing of the manuscript.

## Declaration of Competing Interest

The authors declare no conflicts of interest.
